# Dry eye disease: an introduction

**Published:** 2024-10-02

**Authors:** Nick Astbury

**Affiliations:** 1 Honorary Associate Professor: International Centre for Eye Health, London School of Hygiene & Tropical Medicine, London, UK.

**Dry eye is uncomfortable and can impair sight, reducing quality of life. Early recognition, advice, and treatment can improve symptoms and prevent worsening of the condition.**

The tear film protects, nourishes, and lubricates the eyes, keeping them healthy. It is made of three layers, all of which are important for the eye to function well (Figure 1):

- **The central aqueous (watery) layer.** The bulk of the tear film is made up of watery tears that are formed in the lacrimal glands, are spread over the ocular surface when blinking, and then drain away through ducts on the inner edge of the eyelids and out into the nose, in a continuous cycle.
- **The inner mucous layer.** This is a sticky layer which connects the watery layer of the tear film to the cornea, while allowing it to move freely across the ocular surface, reducing friction and preventing damage when blinking. The mucous layer is produced by cells in the ocular surface.
- **The outer lipid (oily) layer.** The oily outer layer seals the tear film and keeps it stable by reducing evaporation. This layer is also responsible for maintaining a smooth optical surface that allows light to refract through the cornea without distortion. This layer is produced by the meibomian glands in the eyelids.

**Figure 1:**
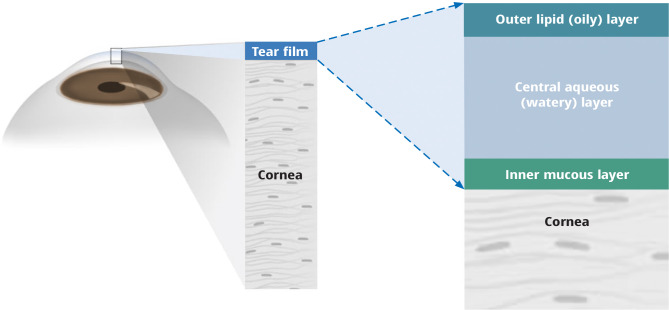
The position and composition of the tear film

© ICEH CC BY-NC-SA 4.0

Without a healthy tear film, eyes become dry, causing discomfort and blurred vision. The tear film can be affected for many reasons.

- **Ageing.** With age, people produce fewer tears, and the tear film composition changes.
- **Blepharitis.** A condition involving blockage of the meibomian glands. Blockage of the glands leads to inflammation and reduced production of the upper oily lipid layer, which increases evaporation of the tear film.
- **Hormonal changes.** Pregnancy or the menopause can alter tear production.
- **Medical conditions.** Diabetes, rheumatoid arthritis, or Sjögren's syndrome can affect the tear film composition, leading to dryness.
- **Medication.** Antidepressants and anti-anxiety tablets can contribute to dry eye. Some eye drops, such as glaucoma medication, can also cause dry eye – especially eye drops that contain preservatives.
- **Contact lenses.** Prolonged wear can worsen dry eye.
- **Environmental exposure.** Air conditioning, smoke, or pollutants can aggravate dry eye disease.
- **Digital screens.** Prolonged use can contribute to dry eye symptoms. This is because people tend to blink less often when looking at screens.

Dry eyes (eyes without a healthy tear film) can become inflamed and there can be damage to the surface of the cornea. If left untreated, a negative cycle can occur: blinking can result in increased friction between the eyelid and cornea, which can damage the cells that produce the mucous layer, further destabilising the tear film and leading to increased evaporation of tears and inflammation of the ocular surface. Eventually, corneal ulcers may develop and eyesight may be lost.

## Symptoms of dry eye

Patients with dry eyes tend to complain of:

- Eye irritation – a gritty sensation or a feeling that their eyes are dry
- Excess mucous secretion – eyes are ‘sticky’, or the person notices dried mucous on waking
- Burning or stinging sensation
- Foreign body sensation
- Blurred vision
- Light sensitivity

## Clinical signs

Some signs of dry eye, such as redness and reduced tear meniscus height, can be seen using magnification and a bright light. A slit lamp is ideal, although it is possible to use the anterior segment loupe of an Arclight or traditional direct ophthalmoscope and a +20 dioptre lens.

**Figure 2:**
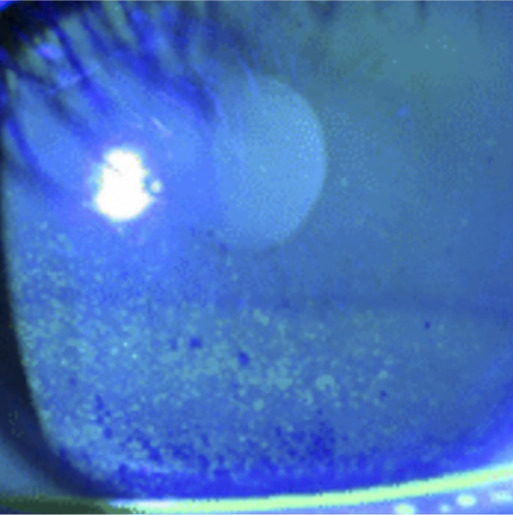
Punctate epithelial erosions.

**Figure 3:**
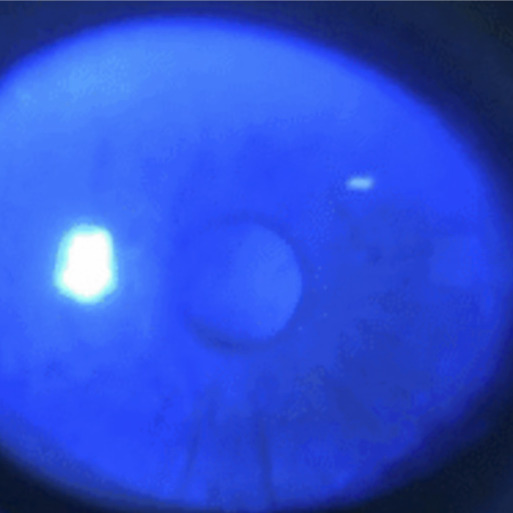
A healthy eye – there are no erosions.

**Figure 4:**
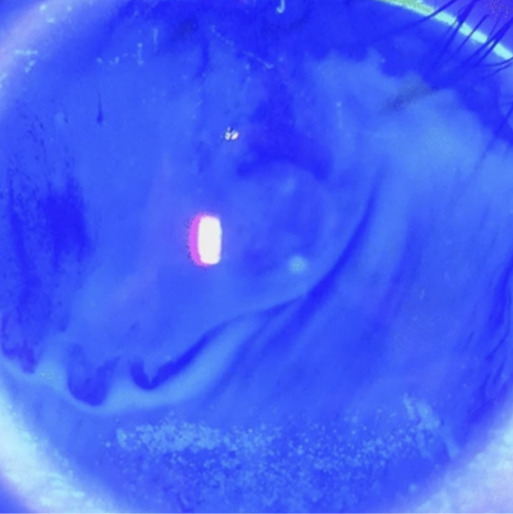
The tear film has started to break up.

**Figure 5:**
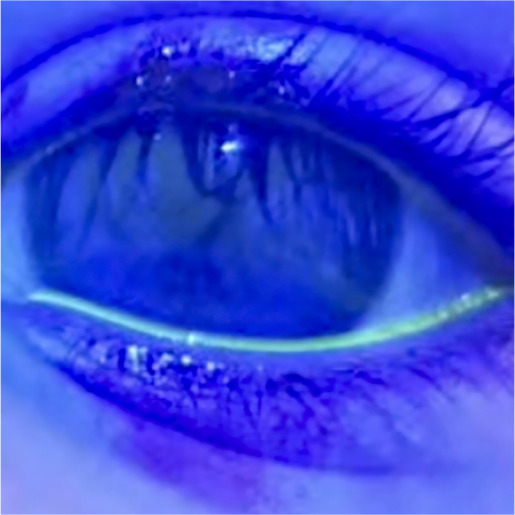
Normal tear meniscus height is 0.2 to 0.3 mm.

The key signs are best seen by instilling fluorescein and examining with either a bright white light or (better yet) a blue light:

1. Look for punctate epithelial erosions (Figure 2), a key sign of dry eye. Figure 3 shows an eye with a healthy appearance; there are no erosions.
2. Observe how long it takes for the tear film to break up after blinking; more than 10 seconds is healthy. Figure 4 shows what it looks like when the tear film breaks up.
3. Estimate the meniscus height: 0.2 mm to 0.3 mm is normal. (see Figure 5); less than 0.2 mm is a sign of dry eye.

© ANDREW BLAIKIE CC BY-NC-SA 4.0

© ANDREW BLAIKIE CC BY-NC-SA 4.0

© ANDREW BLAIKIE CC BY-NC-SA 4.0

© ANDREW BLAIKIE CC BY-NC-SA 4.0

## What to do

All patients with dry eye will benefit from **artificial tears**; preservative-free drops are less likely to worsen the condition. The frequency of use depends upon the severity of the symptoms.

Patients will also benefit from **advice** about avoiding air conditioning, smoke, or other pollutants, reducing the time spent looking at computer or phone screens, and altering their diet by drinking more water and eating foods with omega-3 fatty acids, (such as oily fish, nuts, and seeds) as well as green, leafy vegetables.

Advice about **eyelid care** (warm compresses, eyelid cleaning and massage) can also be helpful first-line treatment, especially in those with blepharitis (see panel).

Symptoms of dry eye can be due to other conditions. If symptoms and signs persist, despite treatment, and there is pain, redness, photophobia, and blurred vision, **refer the person to an eye clinic**. It is important to exclude other causes and to consider different treatments.

The article that follows covers dry eye disease in more detail, including treatments that will not be available in many eye care centres. However, it is helpful to have a thorough understanding of the condition that affects so many people and, in some cases, can be debilitating and cause blindness.

**ADVICE FOR PATIENTS: Lid hygiene to improve meibomian gland function**

Daily eyelid hygiene, such as applying warm compresses, followed by eyelid cleaning and massage, can help to improve meibomian gland function, thereby improving symptoms.

**1. Warm compress**

**Figure 6:**
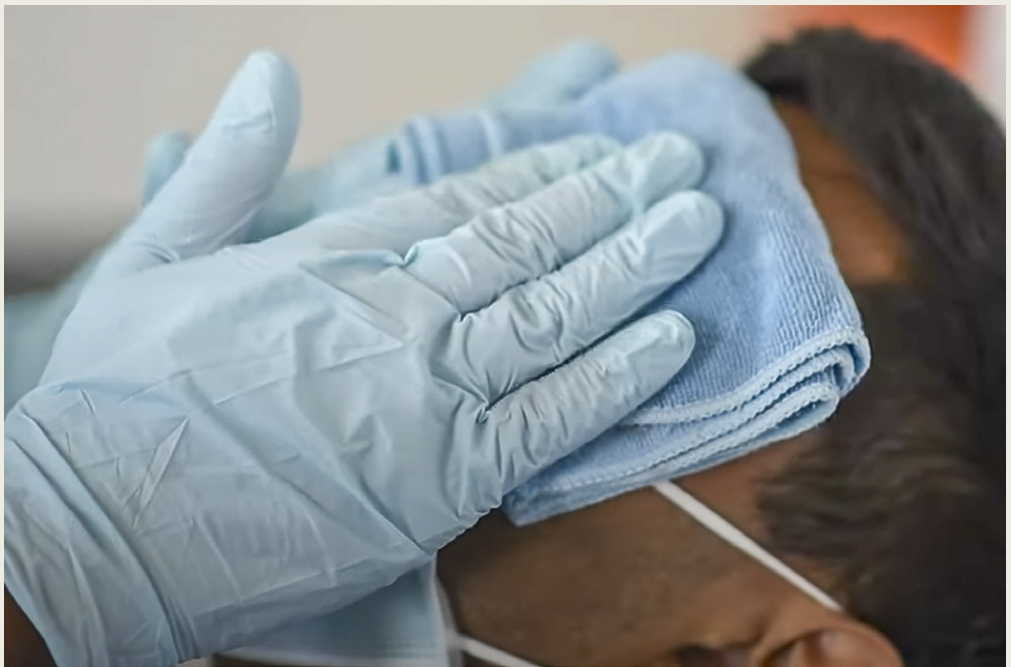
Applying a warm compress to the eyes.

Dip a clean cloth in warm water (not hot), then wring out most of the water and fold the cloth to create a rectangle of approximately 5 cm by 20 cm. Apply gently to the eyelids while lying down or sitting with the head tilted back (Figure 6). Reheat the pad when it cools, so that heat is applied to the lids for a total of 10 minutes. This can help the oily meibomian gland secretions to flow more easily.

© THE ARCLIGHT PROJECT CC BY-NC-SA 4.0

**2. Cleaning**

Use cooled boiled water and a clean face cloth, cotton wool pad, or cotton bud to clean the eyelid margins. Rubbing along the margin can unblock the opening of the glands.

**3. Massage**

**Figure 7:**
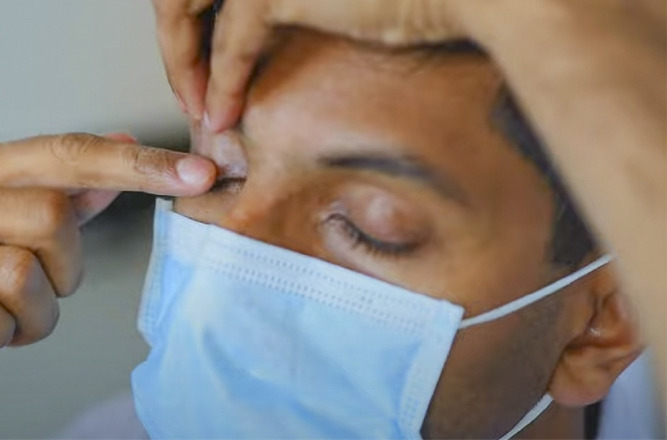
A patient massages their eyelids using a clean fingertip.

Massage the lids towards the lid margin (Figure 7). This can help express the oily meibomian gland secretions, improving dry eye when blepharitis is present.

© THE ARCLIGHT PROJECT CC BY-NC-SA 4.0

This video by the Arclight Project shows how to do this for a patient; patients can also learn to do this for themselves. tinyurl.com/4a8vs66s

